# 

**DOI:** 10.1192/bjb.2017.32

**Published:** 2018-10

**Authors:** John Gossa

**Affiliations:** Student at the University of Central Lancashire and an Associate Specialist in Child and Adolescent Psychiatry with the Priory Group, UK; email: j.gossa@nhs.net

Julia Samuel, grief psychotherapist and Founder Patron of Child Bereavement UK, presents a moving and thoughtful collection of therapeutic encounters. Each brief narrative traces a careful line between study and story, reminiscent of *The Examined Life* by Stephen Grosz. The style of the case story captures the uniqueness of grief and the therapeutic process, reminding us that these are not illustrations of pathology.

Samuel's studies, written in spacious and measured prose, are fearless in their approach. She includes those bereaved by miscarriage, trauma and suicide, and explores the many ways people try to cope. She gently reveals the complex emotional experiences and interpersonal difficulties that emerge, yet her own human response is never far from the text.

The case stories are divided according to the relationship to the deceased, and each section is followed by Samuel's own reflections and references to existing research. The author is keen to present positive accounts of healing and recovery, together with practical advice for the bereaved. This may explain why abnormal grief does not feature among the selected cases.

Samuel's person-centred psychotherapeutic approach is apparent throughout. This alliance carries through to her reflections and may explain why she often does not grapple with conceptual questions or distinctions. Indeed, Samuel avoids an explicit espousal of any particular model of grief. While classical theorists such as Bowlby and Kübler-Ross are summarily acknowledged, Samuel maintains her person-centred focus, helping to construct narratives with her clients. She reveals the tensions that arise and hints at the inner workings of grief while resisting any theoretical speculation. At times, Samuel uses a more integrated therapeutic approach, allowing for a broader understanding of her clients' difficulties according to different modalities. She identifies unhelpful defence mechanisms and cognitive distortions: where grief is bound up in strong cultural identity, Samuel makes reference to Jungian archetypes; to help a client struggling to achieve emotional stability, she works with a transitional object.

The significance of grief may be immediately recognisable to the psychotherapist, whether in the form of bereavement or as part of uncomplicated psychosocial development; however, hidden among Samuel's optimistic reflections are many reminders of why grief matters to the psychiatrist. Historically, grief has represented a line in the sand, demarcating normal experience from psychopathology. However, the removal of the ‘bereavement exclusion criteria’ from DSM-5 appears to challenge this, reintroducing grief as a viable precipitant in some disorders. Additionally, failure to recognise features of normal or unresolved grief can lead to misdiagnosis. Even in cases of uncomplicated grief, hallucinatory phenomena remind us that the boundary between normal experience and mental illness may not be a fixed one. Though choosing to err on the side of hope and resolution, Samuel's tenderly written accounts give voice to the weight of these experiences. Unresolved grief nonetheless continues to raise many questions and, as noted in the DSM-5, remains a recommendation for further study.

